# Transcriptome Analysis of Genes Involved in Fatty Acid and Lipid Biosynthesis in Developing Walnut (*Juglans regia* L.) Seed Kernels from Qinghai Plateau

**DOI:** 10.3390/plants11233207

**Published:** 2022-11-23

**Authors:** Wenjun Shi, Defang Zhang, Zhong Ma

**Affiliations:** Academy of Agriculture and Forestry Sciences, Qinghai University, Xining 810016, China

**Keywords:** walnut, transcriptome sequencing, lipid biosynthesis, fatty acid, gene expression, seed kernel oil

## Abstract

Walnut (*Juglans regia*) is an important woody oil-bearing plant with high nutritional value. For better understanding of the underlying molecular mechanisms of its oil accumulation in the Qinghai Plateau, in this study we monitored walnut fruit development, and 15 cDNA libraries were constructed from walnut seed kernels collected at 72, 79, 93, 118 and 135 days after flowering (DAF). The candidate genes were identified using sequencing and expression analysis. The results showed that the oil content in the kernels increased dramatically in late July and reached the maximum value of 69% in mature seed. More than 90% of the oils were unsaturated fatty acids (UFAs) and linoleic acid (18:2) was the predominant UFA accumulated in mature seed. Differentially expressed genes (DEGs) in 15 KEGG pathways of lipid metabolism were detected. We identified 119 DEGs related to FA de novo biosynthesis (38 DEGs), FA elongation and desaturation (39 DEGs), triacylglycerol (TAG) assembly (24 DEGs), oil bodies (12 DEGs), and transcription factors (TFs, 6 DEGs). The abundantly expressed oleosins, caleosins and steroleosins may be important for timely energy reserve in oil bodies. Weighted gene coexpression network analysis (WGCNA) showed that *AP2/ERF* and *bHLH* were the key TFs, and were co-expressed with *ACC1*, *α-CT*, *BCCP*, *MAT*, *KASII*, *LACS*, *FATA*, and *PDCT*. Our transcriptome data will enrich public databases and provide new insights into functional genes related to the seed kernel lipid metabolism and oil accumulation in *J. regia*.

## 1. Introduction

Walnut (*Juglans regia* L.), as an important woody oil plant, is widely cultivated in many regions around the world. Walnut is rich in several beneficial compounds, e.g., unsaturated fatty acids (UFAs), proteins, minerals, and tocopherols [1], and therefore it has gained tremendous interest due to its nutritional and medicinal benefits [2]. Beneficial effects of walnut consumption have been reported, including anti-atherogenic, anti-inflammatory, antimutagenic, and anticancer effects [3], and protection from diabetes [4,5] or cardiovascular diseases [6]. A mature walnut kernel contains a high oil content, which can vary from 52% to 72% [7,8,9]. The application of vegetable oils is determined by FA composition, which is one of the most important characteristics of vegetable oil quality and which determines the suitability of a vegetable oil for nutrition [2].

Walnut kernels are rich in polyunsaturated fatty acids (PUFAs), which are essential dietary FAs [8]. The FA composition and content of walnut oils in different countries and regions have been reported [2,7,8,9,10]. It has been found that even though walnuts from different areas have the same FA composition, the relative content of each component varies in different production areas [10]. Linoleic acid (LA, 18:2, range 42.5–76%) is the major FA of walnut oil, followed by oleic acid (OA, 18:1, 9–39.2%), linolenic acid (ALA, 18:3, 2–19.2%), palmitic acid (PA, 16:0, 2.9–11.4%) and stearic acid (SA, 18:0, 0.6–2.5%) [8,9,11,12,13,14,15,16]. Walnut species and varieties may affect the FA composition [17]. Environmental factors affect plant growth, development and quality [18]. The growth period of plants is affected by the environment, e.g., with increases in altitude, the plant growth period becomes shorter. It is generally believed that the increase of altitude is conducive to the accumulation of fruit oil content [19,20]. Previous studies report that FA composition and content are affected by growth environments. The responses of different plants to their environment regarding FA content are different [16,19,21,22]. Greve et al. [23] reported that environment, nut maturity, and interactions contribute significantly to variations in PUFA content in walnuts. Gao et al. [15] found that the planting environment was the main factor that affected the composition and content of FAs in walnut oil. The geographical environment also exerts an important influence on the triacylglycerol (TAG) composition and content.

Plant oils, mainly in the form of TAGs, primarily accumulate in seeds and fruits [24]. In plants, the seed oil synthesis process includes de novo FA synthesis and TAG assembly in multiple subcellular organelles [25]. The oil quantity and quality (i.e., FA composition) are controlled and regulated by a series of enzymes that take part in the biosynthesis of all lipids. Recently, new multi-omics technologies, including transcriptomics and metabolomics, have been used to analyze the complex biosynthesis pathway of oils in walnut kernels. Yan et al. [26] found that TGs (18:2/18:2/18:3) and diacylglycerols (DG) (18:2/18:3) were the main glycerolipids, and mapped the main lipid metabolism pathways in walnut. It has been reported that *ACCase*, *LACS*, and acyl-lipid omega-3 desaturase (omega-3 fatty acid desaturase, *FAD7*) are the key genes related to oil synthesis [27]. A total of 104 key genes associated with walnut oil accumulation were obtained from “Xiangling” walnut kernels at three stages. The phospholipid diacylglycerol acyltransferase (PDAT) metabolic pathway may be more conducive to the accumulation of walnut oil. Zhao et al. [28] constructed 16 miRNA–mRNA regulatory modules involved in walnut oil accumulation and FA synthesis. Furthermore, eight known miRNAs and nine novel miRNAs regulate 28 genes related to FA metabolism and lipid synthesis. Huang et al. [29] identified 108 unigenes associated with lipid biosynthesis from RNA sequencing (RNA-Seq) data, and *stearoyl-ACP desaturase* (*SAD*), *delta-12 desaturas*e (*omega-6 desaturase*, *FAD2*), and *omega-3 desaturase* (*delta-15 desaturase*, *FAD3*) were abundantly expressed in the walnut embryo stage. High expression of *FAD2* and *FAD3* is the possible reason for rich PUFAs. A subcellular localization analysis suggested that 18:3 was mainly synthesized in the endoplasmic reticulum (ER) [29]. However, the high contents of oil and PUFAs in walnuts have still not been addressed.

Due to the higher altitude and lower temperatures, the phenological stage of walnuts in the Qinghai Plateau begins later than the lower altitude regions. The synthesis and accumulation of oil is completed in a relatively short time, and the characteristics of gene expression in oil synthesis pathways during seed development are not clear. In this study, the morphological development and changes in fruits, oil content and the transcriptome of walnut seed kernels from the Qinghai Plateau were analyzed to discover the key genes involved in lipid biosynthesis and to dissect the molecular mechanisms of oil accumulation during walnut fruit development. The annotated transcriptome sequences and gene expression profiles provide useful information for the identification of key genes involved in the biosynthesis pathways of lipids and for research on the mechanisms of oil accumulation in walnut kernels.

## 2. Results

### 2.1. Morphological Characteristics and Oil Accumulation during J. regia Embryo Development

**Figure 1:**
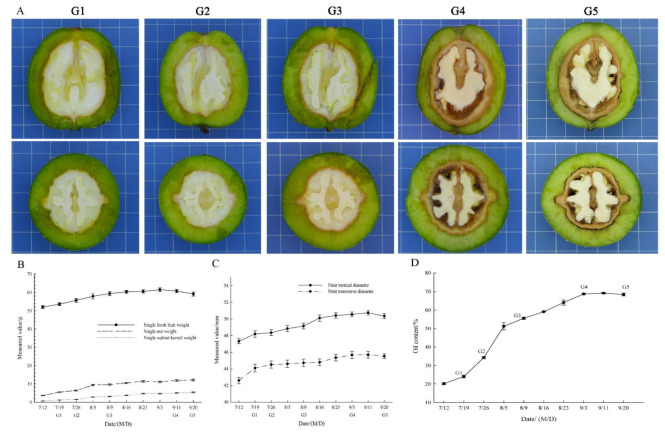
Characterization of walnut fruit and seed kernel development. (**A**) Morphological changes of fruit and walnut kernel growth and development. Walnut fruits were collected on 19 July, 26 July, 9 August, 11 September, 20 September, designated as G1, G2, G3, G4 and G5, respectively. The longitudinal and transverse cuts of walnut fruits are shown in the above and below panels. (**B**) Dynamic changes in single fruit fresh weight, nut and kernel weight of walnuts at different developmental stages. Vertical bars represent standard errors (SE) in all graphs. (**C**) Dynamic changes in the vertical diameter and transverse diameter of the walnut fruits at different developmental stages. (**D**) Dynamic changes in oil content of the walnut seed kernels during seed development.

White cotyledons formed and had clear boundaries with the pericarp (Figure 1A, G1). The walnut shell hardened gradually from the top (G1–G2), and various nutrients such as fat were synthesized and accumulated until the ripening of the fruit. The endocarp was completely lignified at the G4 stage, and the pericarp of the fully mature fruit was readily separated from the seed kernel (G5) (Figure 1A). We began harvesting the walnut fruits on 11 September.

Single fresh fruit weight rapidly increased from 51.983 g to 59.375 g (from 12 July to 9 August), then slowly increased to 61.479 g (3 September), and then slightly decreased from 61.479 g to 59.121 g (from 11 September to 20 September). Single nut weight and single walnut kernel weight showed slow upward trends from 3.63 g to 12.11 g, and 0.72 g to 5.43 g, respectively (Figure 1B, Table S1).

The vertical diameter of walnut fruit slowly increased to 50.73 mm and then decreased to 50.33 mm in the last period; the fruit transverse diameter slowly increased to 45.69 mm and then decreased to 45.52 mm in the last period (Figure 1C, Table S1).

The total oil content of the walnut kernel samples from 10 time points was calculated (Figure 1D, Table S1). The total oil content continuously increased from 20.20% to 69.17% from 12 July to 11 September, and then slightly decreased to 68.43% on 20 September. The total oil content sharply increased from 19 July (24.10%) to 5 August (51.25%), and then showed a slowly rising trend. The maximum oil increase in the walnut kernels appeared between 79 days after flowering (DAF, G2) and 89 DAF (Figure 1D). The mature walnut seed oil content was more than 69% of the total composition of the walnut seed. According to the embryo morphology development and the oil content, seed kernels at the G1–G5 stages were chosen for RNA-seq analysis.

The FA composition and content of walnut oil in the mature walnut seed kernels were analyzed (Table 1), and a total of 16 FAs were detected and measured. There were five kinds of main FAs in the mature walnut seeds: 16:0, 18:0, 18:1, 18:2 and 18:3. The total content of these FAs accounted for more than 99% of the total FAs, while the content of other FAs was lower. The two predominant compositions of saturated fatty acids (SFAs) in mature seeds were 16:0 (6.41 %) and 18:0 (2.04 %), which were maintained at low levels, while the main UFAs were 18:2, 18:1 and 18:3. The total content of the three UFAs accounted for more than 90% of the total FAs. PUFAs were the major FAs, accounting for more than 70%. A proportion of 57.67% of the total FAs was 18:2, and this was the primary fatty acid in walnut oil.

### 2.2. Identification of DEGs and Enrichment Analysis during Seed Development

A total of 709,394,820 raw reads were obtained from 15 sequencing libraries and 103.82 G clean bases were obtained after quality control. All of the clean reads were mapped to the walnut genome sequence; the average total map rate was 97.55% and the proportion of the total map rate ranged from 97.09% to 98.06%. The mapping results of each sample are shown in Table S2. The average reads number mapped to the exon region of the genome was 6,441,750,032 and the average proportion mapped to the exon region of the genome was 95.606%. The mapping results of each sample are shown in Table S3. Over 92% of sequences aligned to the reference genome located in the exon regions. The percentage of the sequences located in intergenic regions ranged from 1.35% to 4.27%; a small proportion of sequences were located in intron regions (Figure S1).

Raw data of all raw reads are available in the NCBI SRA database with the accession number PRJNA781571.

**Figure 2:**
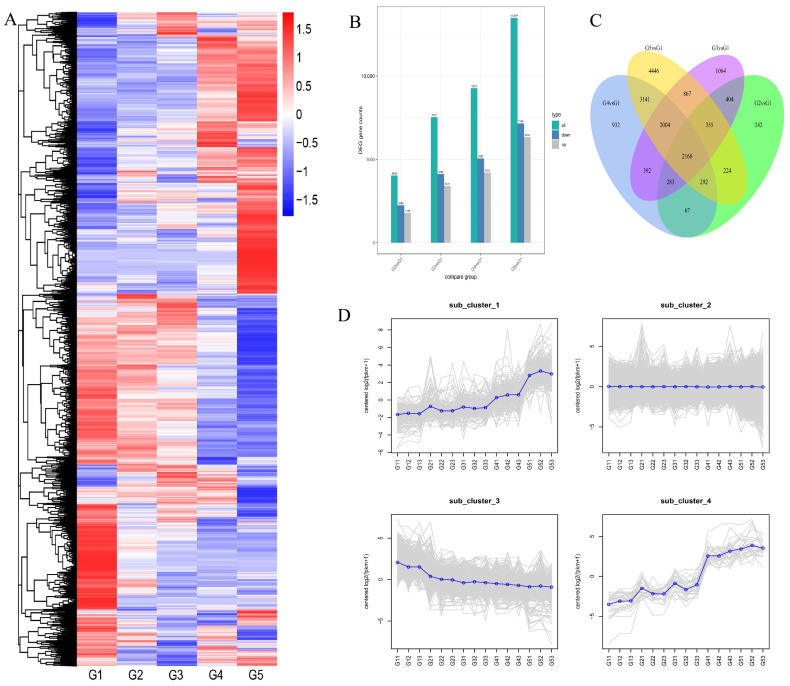
Preliminary analysis of the transcriptome of walnut seed kernels. (**A**) Cluster heat map of differentially expressed genes (DEGs) at five developmental stages. (**B**) Number of DEGs identified in the developing walnut seed kernels compared to G1. (**C**) The Venn diagram of DEGs from four groups. (**D**) The four cluster groups with different gene expression profiles.

The gene expression levels of all samples from five fruit development stages were qualified and compared, and the DEGs were detected. The clustering heatmaps of all DEGs were analyzed (Figure 2A). The numbers of DEGs identified in samples from different developmental stages can be seen in Figure 2B. It is interesting that the numbers of DEGs increased with the walnut seed development. A total of 4035 DEGs were found in G1 vs. G2, including 1789 upregulated genes and 2246 downregulated genes. A total of 7537 DEGs were found in G3 vs. G1, including 3411 upregulated genes and 4126 down-regulated genes; 9279 DEGs were found in G4 vs. G1, including 4219 upregulated genes and 5060 downregulated genes. The most DEGs were found in G5 vs. G1, including 6333 upregulated genes and 7164 downregulated genes. A Venn diagram of DEGs was drawn, and a total of 2168 genes were expressed in all five stages (Figure 2C). To analyze the gene expression pattern, clustering analysis was performed, and all the genes were clustered into four major patterns (Figure 2D).

GO (Gene Ontology) annotation classed all the DEGs. The intracellular organelle parts and organelle parts were the top two terms in G2 and G1, followed by cytoskeletal protein binding, tubulin binding, microtubule binding, microtubule-based process, motor activity, microtubule motor activity, microtubule-based movement, and movement of cell or subcellular component (Figure S2A). Molecular function regulator and cytoskeletal protein binding were the top two terms in G3 and G1, followed by enzyme regulator activity, tubulin binding, motor activity and microtubule binding (Figure S2B). Ribonucleoprotein complex, ribosome, carboxylic acid metabolic process, oxoacid metabolic process and organic acid metabolic processes were the top five terms in G4 and G1 (Figure S2C). Cytoskeletal protein binding, carboxylic acid biosynthetic process, and organic acid biosynthetic process were the top three terms in G5 and G1, followed by tubulin binding, microtubule binding, and pyridoxal phosphate binding (Figure S2D).

**Figure 3:**
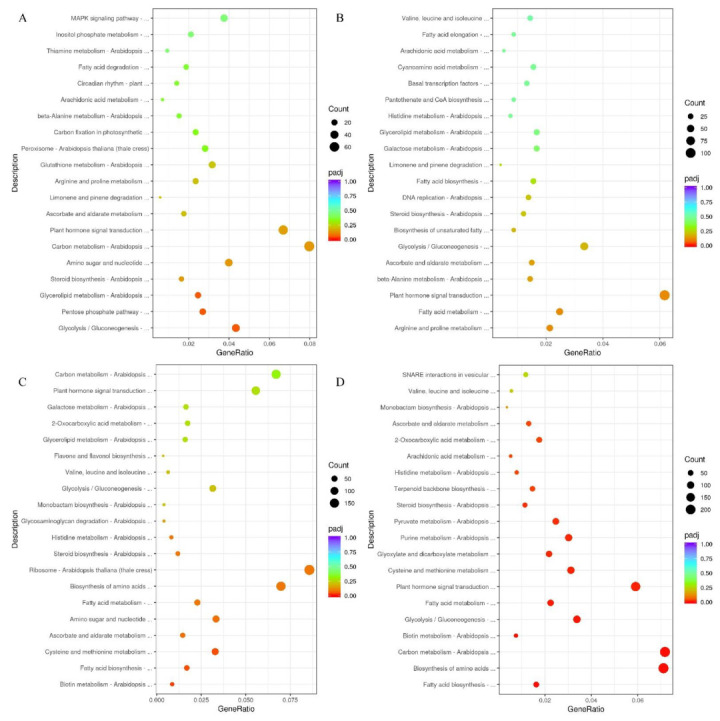
Bubble diagram of enrichment of KEGG pathway. The top 20 enriched KEGG pathways of G2 vs. G1 (**A**), G3 vs. G1 (**B**), G4 vs. G1 (**C**), G5 vs. G1 (**D**). *X* axis represents the enrichment ratio (calculating formula for Rich thewire = Term Candidate Gene Num/Term Gene Num). *Y* axis represents the KEGG pathway, the size of the dot represents the numbers of DEGs. The color of each dot represents the Qvalue; red to purple represent the significance of enrichment.

KEGG (Kyoto Encyclopedia of Genes and Genomes) annotation classed all the DEGs to the pathways, and the top 20 DEGs of the pathways were analyzed (Figure 3). Carbon metabolism (ath01200) was the top pathway that DEGs enriched, followed by plant hormone signal transduction pathway (Ko04075) in G2 and G1 (Figure 3A). Plant hormone signal transduction pathway (Ko04075) was the most enriched pathway in G3 and G1, followed by glycolysis/gluconeogenesis (ath00010) (Figure 3B). Ribosome (ath03010) was the top pathway that DEGs enriched, followed by biosynthesis of amino acid pathway (ath01230) and carbon metabolism in G4 and G1 (Figure 3C). For G5 and G1, the carbon metabolism pathway and biosynthesis of amino acid pathway were the top two pathways, followed by the plant hormone signal transduction pathway (Ko04075) (Figure 3D). There are a few pathways involved in FA biosynthesis and lipid metabolism shown in the figure. A total of 15 pathways of these involved DEGs were annotated to lipid metabolism in our work (Figure S3, Table 2).

### 2.3. Analysis of DEGs for Lipid Metabolism

In addition, the 15 lipid metabolism pathways, including ‘Fatty acid biosynthesis’ (ko00061), ‘Fatty acid elongation’ (ko00062), ‘Steroid biosynthesis’ (Ko00100), ‘Glycerolipid metabolism’ (ko00561), ‘Glycerophospholipid metabolism’ (Ko00564), ‘Arachidonic acid metabolism’ (ko00590), ‘alpha-Linolenic acid metabolism’ (ko00592), ‘Biosynthesis of unsaturated fatty acids’ (ko01040), ‘Fatty acid degradation’ (ko00071), ‘Linoleic acid metabolism’ (ko00591), ‘Sphingolipid metabolism’ (ko00600), ‘Cutin, suberine and wax biosynthesis’ (ko00073), ‘Ether lipid metabolism’ (ko00565), ‘Synthesis and degradation of ketone bodies’ (ko00072) and ‘Fatty acid metabolism’ (ko01212) were selected, to identify and analyze the DEGs. The number of these DEGs in the 15 pathways mentioned above increased with the seed development. In these pathways, there were more downregulated genes than upregulated genes in all comparisons, and the number of DEGs in G5 vs. G1 was the largest. There were 69, 60, 50 and 44 genes involved in fatty acid metabolism, glycerophospholipid metabolism, fatty acid biosynthesis, and glycerolipid metabolism, respectively (Table 2).

### 2.4. Identification and Expression Profiling of DEGs for Fatty Acid Biosynthesis, Elongation, and Desaturation

**Figure 4:**
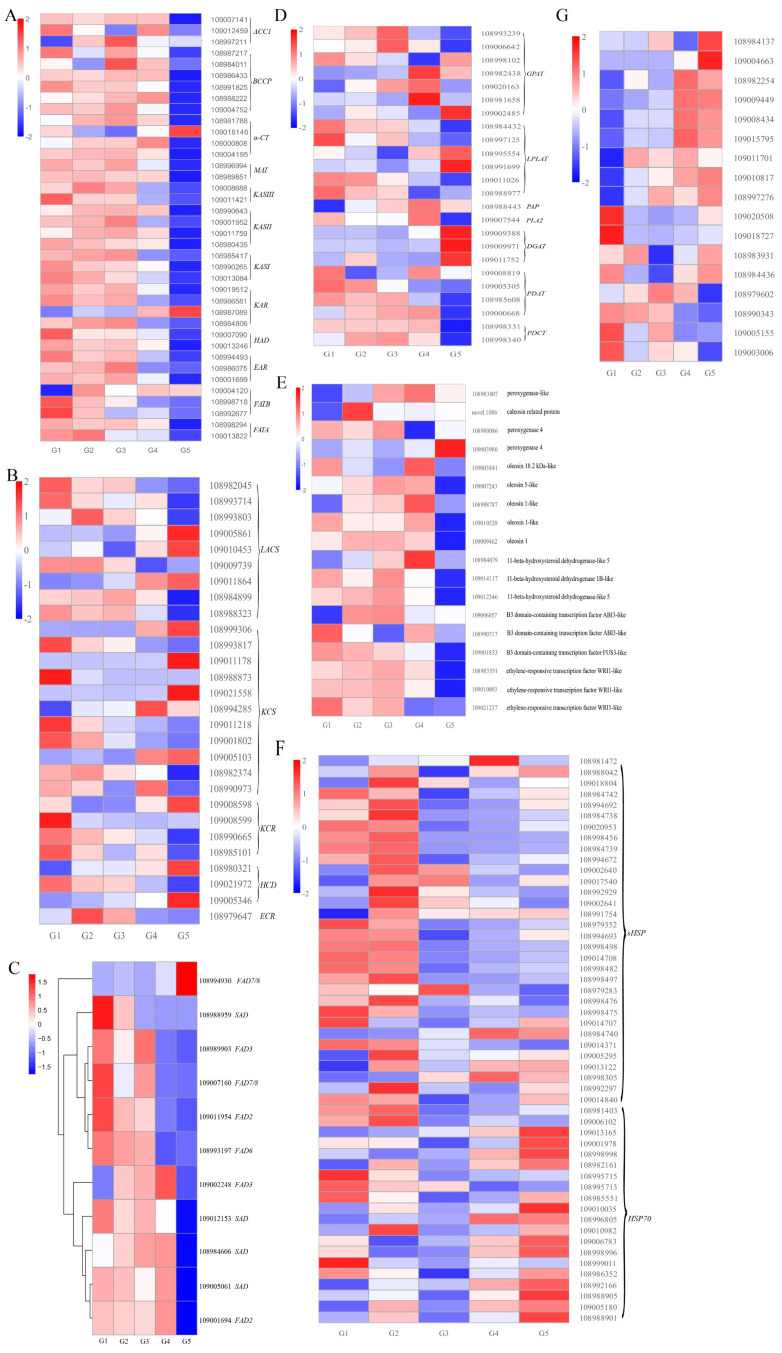
Heat maps of lipid biosynthesis, oil body and TF genes in the developing walnut seed kernels. (**A**) Expression patterns of DEGs related to fatty acid biosynthesis. (**B**) Heat map of DEGs involved in fatty acid elongation. (**C**) Heat map of DEGs involved in fatty acid desaturation. (**D**) Heat map of DEGs involved in TAG assembly. (**E**) Heat map of DEGs related to oil body. (**F**) Heat map of differentially expressed *HSP* genes. (**G**) Heat map of differentially expressed *HSF* genes. Abbreviations: ACCase, acetyl−CoA carboxylase; BCCP, biotin carboxyl carrier protein; α−CT, α−carboxyltransferase; ACP, acyl carrier protein; MAT, malonyl−CoA ACP S−malonytransferase; KAS, 3−oxoacyl−ACP synthase; KAR, 3−oxoacyl−ACP reductase; HAD, 3−hydroxyacyl−ACP dehydratase; EAR, enoyl−ACP reductase; FAT, fatty acyl−ACP thioesterase; LACS, long chain acyl−CoA; KCS, 3−ketoacyl−CoA synthase; KCR, very−long−chain 3−oxoacyl−CoA reductase; HCD/PAS, very−long−chain (3R)−3−hydroxyacyl−CoA dehydratase; ECR, very−long−chain enoyl−CoA reductase; SAD, stearoyl−ACP desaturase; FAD, fatty acid desaturase; GPAT, glycerol−3−phosphate acyltransferase; LPLAT, lysophospholipid acyltransferase; PAP, phosphatidate phosphatase; PLA2, phospholipase A2; DGAT, diacylglycerol O−acyltransferase; PDAT, phospholipid: diacylglycerol acyltransferase; PDCT, phosphatidylcholine:diacylglycerol cholinephosphotransferase; TAG, triacylglycerol.

In this study, we focused on the key genes associated with lipid synthesis and oil accumulation. Based on the gene annotation for the transcriptome of the five developmental walnut seeds, DEGs related to FA biosynthesis, FA elongation, biosynthesis of UFAs, TAG biosynthesis, oil body and transcription factors (TFs) were screened to further investigate their expression patterns during walnut seed development (Figure 4).

Acetyl-CoA carboxylase (ACCase) catalyzes the first step of the FA biosynthesis pathway in the plastids. Acetyl-CoA conversion to malonyl-CoA is catalyzed by ACCase that consists of four subunits, including biotin carboxylase (BC), carboxyl transferase subunit alpha (α-CT), carboxyl transferase subunit beta (β-CT) and biotin carboxyl carrier protein (BCCP). A total of 13 DEGs encoding *ACCase* and its subunits, including three *ACC1*, six *BCCP*, and four *α-CT,* were found in this research. Our analysis revealed that *ACC1* (109007141), *BCCP* (108991825, 108988222) and *α-CT* (109000808) were the dominant ones because their transcriptional levels were maintained at high levels (KPFM > 80) from G3 to G4, and then reduced afterwards at the G5 stage. This may promote rapid increases in oil content during this stage. The transcriptional levels of six *BCCP* repeats were similar, and were high at the G1–G4 stage and lower at the G5 stage. 

Among the four *α-CT*, three *α-CT*s (108981788, 109000808 and 109004195) showed a similar pattern which was upregulated continuously from the G1 to G4 stage, but downregulated at the G5 stage. The transcription of the other *α-CT* (109018146) was maintained at a low level during G1-G4, but was high at the G5 stage. Then, MAT further transfers malonly-CoA to malonly-ACP. Two DEGs was identified as *MAT*, were highly expressed from G1 to G4, and showed the lowest level at the G5 stage. Subsequently, six continuous condensation reactions are catalyzed by KAS, KAR, HAD and EAR. The 14:0-ACP is transformed to 16:0-ACP, after seven cycles of those reactions, 4:0-ACP is transformed to 18:0-ACP. Then, 16:0-ACP and 18:0-ACP are transformed to 16:0 and 18:0 by FATB and FATA, respectively. We identified 23 DEGs, including two *KASIII*, four *KASII*, three *KASI*, four *KAR*, two *HAD*, three *EAR*, three *FATB*, and two *FATA* (Figure 4A, Table S4). Among these DEGs, one *KAR* (108987089) and one *FATB* (109004120) showed similar transcriptional patterns, and their transcriptional levels were the lowest at G1 and higher from G2 to G5. The transcription of the other DEGs mentioned maintained high levels from G1 to G4, but had the lowest level at G5. These expression patterns provided an explanation for the continuous and rapid oil accumulation during early seed developmental stages (G1 to G4). 

FAs (C ≥ 16) are transformed to long-chain acyl-CoA (C ≥ 16) by LACS and transported to the ER. Subsequently, long-chain acyl-CoA (C ≥ 16) is transformed to long-chain acyl-CoA (C + 2) by KCS, KCR, HCD/PAS2 and ECR. A total of 9, 11, 4, 3, and 1 DEGs were identified as *LACS* (2 *LACS1*, 1 *LACS4*, 2 *LACS6*, 1 *LACS*7, 1 *LACS8*, 2 *LACS9*), *KCS* (*KCS1*, *KCS2, KCS4,* 2 *KCS6, KCS10,* 4 *KCS11, KCS12*), *KCR*, *HCD* (*HCD*, 2 *PAS2A*) and ECR, respectively (Figure 4B, Table S4). Among these genes, the transcriptional levels of *LACS6* (109005861, 109010453), *LACS8* (109011864), *HCD/PAS2* (109005346), *LACS9* (108984899), *KCS10* (108994285), and *KCR* (108990665) were high, and the first four genes reached their peak at G5. *LACS9* and *KCS10* had the highest expression at G3 and G4, respectively.

UFAs were the major FAs in walnut oil. The biosynthesis of UFAs through the plastid pathway or endoplasmic pathway starts with 18:0-ACP. The first pathway, the 18:0-ACP, can be desaturated to oleoyl-ACP (18:1-ACP) by SAD in the plastid, and then 18:1-ACP is dehydrogenized to linoleoyl-ACP (18:2-ACP) by delta-12 desaturase (FAD6, chloroplastic type) in the plastid. Subsequently, under the effect of delta-15 desaturase (FAD7/8, chloroplastic type), 18:2-ACP is transformed to 18:3-ACP in the plastid. In the second pathway, 18:1-ACP is hydrolyzed to free 18:1 by the FATA. Free fatty acids (16:0, 18:0, 18:1) are esterified to FA-CoA by LACS and then transported into ER [25]. Next, 18:1-CoA and lysophosphatidylcholine (LPC) are transformed to 18:1-PC by lysophosphatidylcholine acyltransferase (LPCAT), and in turn are dehydrogenated to 18:2-PC and 18:3-PC by FAD2 and FAD3 [30,31]. In this study, four, two, three, one and two DEGs were identified as *SAD, FAD2*, *FAD3*, *FAD6* and *FAD7/8*, respectively. There were no DEGs identified as FAD5. The transcriptional levels of *SAD*, *FAD2* and *FAD3* were high, while *FAD6* and *FAD7/8* were less expressed in developing walnut seeds. The expression heat map showed that *SAD* (108984606, 109005061 and 109012153), *FAD2* (109001694) and *FAD3* (109002248) were grouped into one category, with high expression levels at G1−G4 and the lowest expression levels at G5 (Figure 4C, Table S4). Moreover, *SAD* (108984606, 109005061), *FAD2* (109001694) and *FAD3* (109002248) had the highest expression at G4, and FPKM had values of 1121.26, 778.72, 1675.20 and 4629.80, respectively (Table S4). The transcriptional levels of *SAD* (109012153) reached their peak at G1. Among the four repeats of *SAD* in walnut, *SAD*s (108984606, 109005061) were remarkably transcribed (FPKM > 140), followed by *SAD* (109012153). The expression pattern of these genes suggested that the highly expressed *SADs* rapidly catalyzed the synthesis of UFAs upstream of lipid biosynthesis in the plastids, while the highly expressed *FADs* catalyzed the synthesis of UFAs downstream in the ER. 

### 2.5. Identification and Expression Profiling of DEGs for TAG Assembly and Oil Accumulation

TAG assembly takes place in the ER and is synthesized by the acyl-CoA-dependent pathway (Kennedy pathway) and the acyl-CoA-independent pathway. Glycerol-3-phosphate (G3P) and acyl-CoAs are taken as primary substrates [32]. Glycerol-3-phosphate acyltransferase (GPAT), lysophospholipid acyltransferases (LPLATs), phosphatidic acid phosphatase (PAP), phospholipase A2 (PLA2), DGAT, LPCAT, PDAT and phosphatidylcholine: diacylglycerol cholinephosphotransferase (PDCT) are involved in TAG assembly. Seven, six, one, one, three, four and two DEGs were identified as *GPAT*, *LPLAT*, *PAP*, *PLA2*, *DGAT, PDAT* and *PDCT*, respectively. Among the seven *GPAT*s, *GPAT3* (109006642) exhibited higher expression levels than the other *GPAT*s during seed development, with FPKM values ranging from 19.15 to 39.04, reaching a peak at G3 and then significantly decreasing. The expression levels of *GPAT*s (108982438, 109020163 and 108981658) decreased significantly after attaining a peak at G4. One *PAP* and one *PLA2* were highly expressed at G3, with FPKM values of 12.11 and 132.92, respectively, and which then decreased. LPLAT superfamily members are acyltransferases of de novo and remodeling pathways of glycerophospholipid biosynthesis. The incorporation of an acyl group from either acyl-CoAs or acylACPs into acceptors such as glycerol 3-phosphate, dihydroxyacetone phosphate was catalyzed by the proteins mentioned above. LPLATs such as LPCAT-1, lysophosphatidylethanolamine acyltransferase (LPEAT, also known as MBOAT2) are included in this superfamily [33,34,35]. The FPKM (fragments per kilobase of transcript sequence per millions base pairs sequenced) values of six *LPLATs* ranged from 0.73 to 124.65, of which the FPKM of *LPLAT* (108984432) was higher (24.82 to 124.65), while the remaining DEGs expression levels were relatively low. *LPLAT*s (108984432, 108997125, 109011026 and 108988977) were highly expressed at G1, and the others (108995554, 108991699) were highly expressed at G5 (Figure 4D, Table S4).

The expression levels of *PLA2*s were high during all the developmental stages, with FPKM values from 65.72 to 132.92. The expression levels of *PDCT* (108998331) were maintained at a stable, high level (78.49~89.29) at G1–G4, and then significantly declined to a low level (FPKM < 9). Another *PDCT* was less expressed during the seed development, with FPKM values from 0.16 to 2.09. DGAT and PDAT are essential enzymes for TAG biosynthesis. In walnuts, among the three *DGAT1s*, the expression level of *DGAT1* (109011752) was significantly higher than that of other members during seed development (FPKM > 27), while the transcription of one *DGAT1* (109009971) was maintained at a low level all along (FPKM < 1). The expression levels of the three *DGAT1s* were most abundant at G5. The two *PDAT*s (109000668, 109008819) exhibited higher expression levels than others during the seed development (FPKM > 12), and one *PDAT* (109000668) was upregulated continuously at G1–G4, then downregulated at G5. These results suggested that TAG assembly occurred during the entire seed development (G1–G5) under the synergistic effect of these genes (Figure 4D, Table S4).

Seed oils are mainly stored in the form of TAG, which is stored in oil bodies (OBs). Oleosins (OLEs), caleosins (CLOs) and steroleosins (STEs) are associated with seed OBs. Twelve DEGs (four *CLO*, five *OLE* and three *STE*) were identified in this work. Throughout development, the five gene homologs encoding *OLE* were highly expressed in the seed kernel (595 < FKPM < 7678) (Figure 4E, Table S4), and had the highest expression levels at G3 or G4. In addition, the *OLE*-encoding genes were more strongly upregulated than the *CLO*- or *STE*-encoding genes from G1 to G4. Among the four *CLOs*, *CLO* (10893007) exhibited higher expression levels than other *CLOs* (FPKM > 573), reached a peak at G4, and then declined at G5. The expression level of *STE* (108984079) was significantly higher than the other *STEs* and was expressed to the highest level at G4. TFs are important activators which modulate gene expression at the transcriptional level. Besides the lipid-related genes, six crucial TFs were differentially transcribed during the seed development, including two B3 domain-containing transcription factor ABI3 (ABI3), one B3 domain-containing transcription factor FUS3 (FUS3), and three ethylene-responsive transcription factor WRI1 (WRI1). The two *ABI3*s were highly expressed at G1–G5. The differentially transcribed *WRI1* (109021237) maintained a relatively low level at G1–G5 (FKPM < 1). *FUS3* and *WRI1* (108983551, 109010003) were highly expressed at G1–G4, but declined sharply at the following stage. 

### 2.6. Identification and Expression Profiling of DEGs of HSPs and HSFs

Because of its unique geographical location, in the Qinghai Plateau there are large temperature differences between day and night (Figure S4). Temperatures are typically lowest in the morning and reach a maximum in the afternoon. There may be a protective mechanism during seed kernel development to eliminate the impacts of large temperature differences and protect seed development and the accumulation of oil. Interestingly, differentially expressed *HSP*s were found in developing seeds, and they were identified as *HSP70* and small *HSPs* (*SHSPs*). Many *HSP*s were highly expressed at G1, G2, and G5 (Figure 4F, Table S4), which may be caused by the large fluctuation of day and night temperature. In addition, 17 DEGs were identified as heat stress TFs or heat shock factors (*HSFs*). The transcriptional level of most of the *HSFs* had a relatively low level at G3 (Figure 4G, Table S4).

### 2.7. Validation of RNA-Seq Results by Quantitative Real-Time PCR (qRT-PCR)

**Figure 5:**
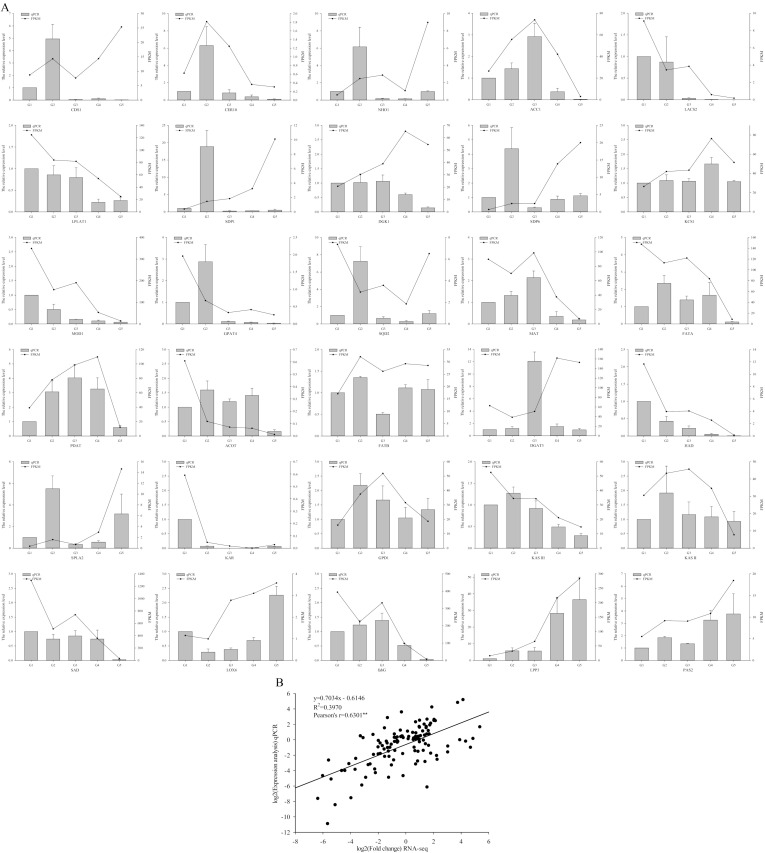
qRT−PCR validation of DEGs related to lipid biosynthesis and metabolism in walnut. (**A**) Comparison of the expression levels determined by RNA−seq and qRT−PCR. Symbols represent mean values and short vertical lines indicate SE (n = 3). (**B**) Correlation analysis of the RNA−seq (FKPM) and qRT−PCR (2^−∆∆ct^) results. The results were calculated using a log2 fold−change measurement. Pearson’s r indicates the Pearson correlation coefficient. ** indicates 0.01 significance.

The expression level and temporal transcription patterns of 30 DEGs (Table S5) involved in lipid biosynthesis and metabolism were analyzed to verify the accuracy of our RNA-seq data. Figure 5A shows the expression profiles of the 30 genes related to lipid biosynthesis and metabolism. The expression patterns of the DEGs (CER10, ACC1, LPLAT1, KCS1, PDAT, FATB, HAD, KAR, GPD1, KAS II, LOX6, LPP3 and PAS2) detected by qRT-PCR were consistent with those estimated by RNA-seq (Figure 5A). Correlation analysis between the RNA-seq and qRT-PCR expression levels of DEGs was conducted (Figure 5B). The expression levels of the DEGs were positively correlated (*p* < 0.01) between the RNA-seq and qRT-PCR results, and the Pearson correlation coefficient was 0.6301 (Figure 5B).

### 2.8. Weighted Gene Co-Expression Network Analysis 

**Figure 6:**
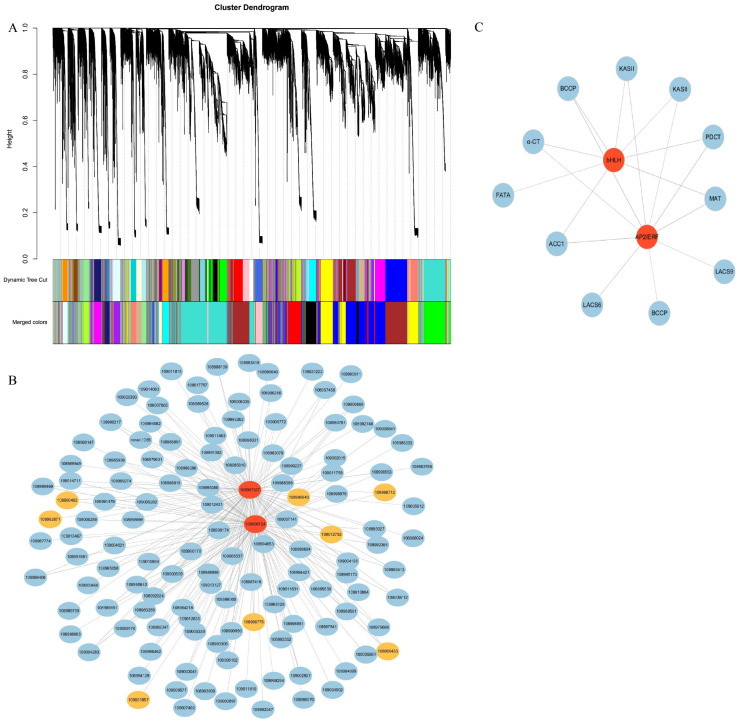
WGCNA of genes in developing walnut seed kernels. (**A**) Gene co-expression modules detected by WGCNA. The clustering dendrogram of the genes across all the samples exhibits dissimilarities based on topological overlap, together with the original module colors (dynamic tree cut) and assigned merged module colors (merged dynamic). (**B**) Primary co-expression network for hub genes for *AP2/ERF* (109006724) and *bHLH* (108987327). Blue ellipses represent gene, yellow ellipses represent TF. (**C**) Co-expression network between TFs and oil biosynthesis communication-related genes in blue module. Red ellipses represent hub genes. The edge width represents the weight value between the two nodes.

Weighted gene co-expression network analysis (WGCNA) was carried out in order to study the relationship between the DEGs and the oil content in walnut seed kernels. The results indicated that 35 modules were identified and marked with different colors (Figure 6A, Figure S5A). The module–trait correlation relationships were analyzed, which showed that the blue module had the highest correlation with oil content of *J. regia* (Figure S5B,C). The most abundant TF families in the blue module were AP2/ERF (36), WD40 (32), MYB (30), bHLH (16), bZIP (16), NAM (14), BTB (13), and WRKY (12) (Table S6).

Gene co-expression analysis indicated that the TFs *AP2/ERF* (109006724) and *bHLH* (108987327) were the hub genes of the blue module (Figure 6B). Gene expression analysis showed that the expression trends of the two TFs were similar with those of the genes related to oil biosynthesis, that is, were highly expressed at G4–G5 (Figure S5D). Moreover, *AP2/ERF* (109006724) and *bHLH* (108987327) were co-expressed with genes related to oil biosynthesis, such as *ACC1* (109007141), *α-CT* (109004195), *BCCP* (108988222, 108986433), *MAT* (108989851), *KASII* (109011759, 108990643), *LACS* (109005861, 108984899), *FATA* (108998294), and *PDCT* (108998331) (Figure 6C).

## 3. Discussion

Walnut (*J. regia*) is an important oil-bearing plant with high nutritional value. Recently, RNA-seq has been shown to be one of the effective methods of revealing the biological mechanism of lipid metabolism and oil accumulation in the oilseed crops. In our study, the oil contents of walnut seed kernels at different developmental stages growing at high altitude were analyzed. DEGs and TFs related to lipid biosynthesis and metabolism were screened and obtained through transcriptomics analysis. 

In this study, the walnuts flowered in early May in the Qinghai Plateau, and the fruit was harvested in mid-September. The time from full blossom to fruit maturity was about 126 days. The walnut fruit growth period in the Qinghai Plateau in this research is shorter than that in Xinjiang, which was about 150 days in a site 1394 m above sea level [36]. The plant growth period is shorter with the increase in altitude. The sampling site of this study is at an elevation of 2102 m, and there is also a large temperature difference between day and night (Figure S4). Therefore, we believe that the plateau environment led to the shorter growth period in this study. The fruit development, single fruit weight, nut weight, kernel weight, and fruit vertical and transverse diameters showed increasing trends in general (Figure 1B,C). The single fruit weight and the fruit vertical and transverse diameters slightly decreased after reaching maximum values in the late developmental phase (G5), due to the moisture content decreasing during fruit development, accompanied by the decrease in single fruit weight and the fruit vertical and transverse diameters caused by dehydration and shrinkage of seed and pericarp. 

In the process of walnut fruit development, the oil content showed a trend of slow–fast–slow. The oil accumulated slowly in the early stages of fruit development, then accumulated rapidly at 70 to 110 DAF. Thereafter, the oil accumulated slowly until fruit ripening [37]. The oil bodies were first observed at 60 DAF in the embryo, and then the number of oil bodies gradually increased until the fruit ripening [36]. In the present study, the oil content increased gradually during fruit development and maturation, which was consistent with previous studies [37] on the dynamic accumulation of walnut oil. The mature walnut nut was made up of more than 69% oil. The FA composition of the mature walnut oil was further investigated, and we found more than 90% were UFAs and more than 70% were PUFAs (Table 1). The contents of the two predominant compositions of SFAs were maintained at low levels. These results showed that the walnut kernel contained more PUFAs and fewer MUFAs, which was similar to previous descriptions of walnuts cultivated in other regions in China and in other countries (Italy [16], Turkey [38], New Zealand [11]). Therefore, walnuts from the Qinghai Plateau are good edible oil sources with high amounts of PUFAs. However, Geng et al. [39] reported that MUFA content was the highest in walnuts from Yunnan. Previous studies showed that FA composition and content are affected by growth environments [40,41]. It has been found that the content of 18:3 in soybean was the most vulnerable to environmental changes [41]. Temperature is one of the essential environmental factors affecting plant metabolism. Therefore, we speculate that the temperature of the sampling site affected the UFA content. In this study, HSPs were highly expressed at G1, G2, and G5, and this may be caused by the large fluctuation of day and night temperature in Qinghai. How walnut plants adapt to large diurnal temperature fluctuations to protect seed development and oil accumulation needs more research in the future. 

In this study, 15 cDNA libraries for transcriptome sequencing of walnut seed kernels at five developmental stages were constructed, more than 97% of clean reads in each library mapped to the walnut reference genome. We obtained four sets of DEGs between the different seed development stages. There were more downregulated genes than upregulated genes in lipid synthesis pathways. We focused on FA biosynthesis and oil accumulation in the developing walnut seed kernels, which involves FA biosynthesis, TAG assembly and lipid storage.

The 18C UFAs are important constituents of vegetable oils (e.g., camellia, hickory, walnut), and the regulatory mechanisms are different among plants. With dehydrogenation by SAD, 18:0-ACP is transformed to 18:1-ACP in the plastid, and then 18:1-ACP is transformed to 18:2-ACP and 18:3-ACP in the plastid by FAD6 and FAD7/8, respectively. In another pathway, 18:1-ACP is transformed to 18:1-PC by a series of enzymes, which is desaturated by FAD2 and FAD3 to form 18:2-PC and 18:3-PC in the ER. The results of studies on mutation, overexpression, or heterotopic expression of these genes showed that they have an important influence on oil accumulation or FA compositions. The 18:1 FA is dominant in *Camellia oleifera* and *Carya cathayensis* Sarg. [42,43], and the perfect coordination of high *SAD* levels with low *FAD2* levels enhanced the accumulation of 18:1. High 18:2 accumulation in the seeds of *Artemisia sphaerocephala* and *Gossypium hirsutum L.* is due to the high expression of *FAD2* and the low expression of *FAD3* [44,45]. Previous studies indicated that the expression of FAD3 and FAD7/8 are variable in plants with high 18:3 content. The upregulation of *FAD3* was associated with 18:3 accumulation in seeds of *Paeonia ostii* and *Linum usitatissimum* [46,47]. In addition, the accumulation of 18:3 was associated with the expression of *FAD2* and *FAD3* in *Perilla frutescens* seeds [48].

It has been reported that *J. regia* L is rich in PUFAs, which may be due to high expression levels of FAD2 and FAD3 [29]. The results of subcellular localization confirmed that JrFAD3 plays a part in the ER rather than the plastid [29]. However, what causes high 18:2 and low 18:3 is still unknown.

In this study, UFAs accounted for more than 90% of the total FAs, and more than 70% were PUFAs (Table 1). Four, two, three, one and two DEGs were identified as SAD, FAD2, FAD3, FAD6 and FAD7/8, respectively. The transcriptional levels of *SAD*, *FAD2* and *FAD3* were high, while *FAD6* and *FAD7/8* were less expressed in developing walnut seeds, which was consistent with previous results [29]. In our work, the expression levels of *SAD, FAD2,* and *FAD3* were high from G1 to G4 (Figure 4C), and reached their peak at G4. This trend is consistent with oil accumulation, where the oil continuously and rapidly increased at G1–G4. However, the expression levels of FAD7/8 (108994930) were less than one during walnut seed development (Figure 4C, Table S4). Based on this, we speculated that 18:2 and 18:3 in walnut seeds were mainly produced by the highly expressed FAD2 and FAD3 in the ER rather than by FAD6 and FAD7/8 in plastids. 

DGAT and PDAT are essential enzymes for TAG biosynthesis, and their contributions to TAG assembly varies in different species. Studies show that *PDAT* exhibits higher expression levels than *DGAT* in *Carthamus tinctorius* L., *G. hirsutum* L., *J. regia* L., and *P. ostia.* This indicates that *PDAT* might play a more important role in the process of TAG biosynthesis [29,44,46,49]. In *Torreya grandis* kernels, PDAT showed a higher correlation with oil content than DGAT, indicating that PDAT contributed more to the accumulation of oil than DGAT [50]. However, it was found that DGAT was more important for the biosynthesis of TAG in many oilseeds, such as *Brassica napus* and *Paeonia lactiflora* [51,52]. Furthermore, *PDAT* and *DGAT* simultaneously regulated TAG biosynthesis in *A. sphaerocephala* [45]. The expression level of *PDAT* in walnut is much higher than that of *DGAT1* and *DGAT2*, and *PDAT* is highly expressed at 63−133 DAP [29]. From this study, two *PDATs* (109000668, 109008819) exhibited higher expression levels than the others during the seed development. One *PDAT* (109000668) was upregulated continuously at G1–G4 then downregulated at G5. This expression trend of *PDAT* was similar to that of *PDAT* reported by Huang et al. [29]. Further, in our work, the expression level of *DGAT* (109011752) was much higher than that of the other two *DGAT*s (109009388, 109009971) during seed kernel development, and reached a peak at G5. These results indicated that it might be that PDAT and DGAT simultaneously regulate TAG biosynthesis in walnut.

It has been reported that oleosins controlling oil body structure and lipid accumulation are important proteins in seed [53]. In this study, the *OLE*s were highly expressed from G1 to G4, and were expressed at much higher levels than the *CLOs* and *STEs.* The expression trend of *OLE*s, *CLO* (10893007) and *STE* (108984079) were consistent with oil accumulation. Thus, they might play an important role in lipid accumulation, and high expression levels of *OLE*s may be closely related to high oil content of walnut kernels.

Previous studies have shown that TFs, such as LEC, WRI1, FUS3 and ABI3, play roles in seed oil synthesis and deposition, but this varies from species to species. WRI1 and NF-YB6 were considered to be elite TFs in the regulation of lipid metabolism in *G. hirsutum* L. [44]. WRI1 and FUS3 may be crucial in the regulation of lipid biosynthesis in the kernel of *P. ostii* and *T. grandis* [46,50]. In walnut cultivar “Linzaoxiang”, the expression pattern of WRI1 aligned with the accumulation of oil, and WRI1 may play an important role in the synthesis of walnut oil [29]. Wang et al. [54] identified five important TFs (WRI1, ABI3, FUS3, PKL and VAL1) which might highly regulate ACCase, KASII, LACS, FAD3 and LPAAT. In this study, the results of gene co-expression analysis showed that AP2/ERF and bHLH were the key TFs for the walnut oil accumulation during seed kernel development.

The differential activity of one or more enzymes in each step might lead to the variation in the oil content in developing walnut seeds. Thus, it would be extremely important to study the genes related to oil content and FA composition and content of walnut seed varieties, in respect to copy numbers, allelic combination, transcriptional and post-translational regulations [48]. The oil synthesis is a complicated process that involves a series of enzymes and molecules. Some important genes differentially expressed in the developing walnut seed kernels were identified. Additionally, some TFs that might be related to the FA biosynthesis and oil accumulation of *J. regia* were identified. Nevertheless, the regulating mechanism of oil accumulation in developing seed kernels is still unclear. Further studies on their functions in FA biosynthesis and oil accumulation are required.

## 4. Materials and Methods

### 4.1. Plant Materials

All of the fruits were collected from a local walnut variety (30-year-old walnut trees), which is located in Jianzha county (101°57′ E, 36°01′ N, elevation 2102 m, Qinghai province, China). The annual average temperature is 8.3 °C, the annual average precipitation is 350~400 mm, and the annual sunshine hours are around 2500 h.

### 4.2. Sampling and Fruit Growth Analysis

Samples were collected every 7~10 days during the seed development in 2019. The fruits were collected from areas with the same sunlight conditions. Walnut fruits were harvested on 12 July, 19 July, 26 July, 5 August, 9 August, 16 August, 23 August, 3 September, 11 September, and 20 September (65, 72, 79, 89, 93, 100, 107, 118, 126, and 135 DAF, respectively). According to the results of the pilot study, due to the low oil extraction rate at the early stages, the oil content was continuously analyzed from early July to mid-September. According to the embryo morphology development and oil content, the seed kernels collected at 72, 79, 93, 118 and 135 DAF (designated as G1, G2, G3, G4 and G5, respectively) (Figure 1A) were chosen for RNA-seq analysis. The walnut husk and hull were removed quickly, and the peeled walnut kernel samples for transcriptome sequencing and qRT-PCR were immediately frozen in liquid nitrogen and then taken back to the laboratory, and stored in a refrigerator at −80 °C until use. All of the samples were collected with three biological replicates. At the same time, the vertical and transverse diameter of walnut fruit were measured using vernier calipers. One percent electronic balance was used to determine the weight of fruit, nut, and seed kernel. The mean values of 20 fruits were calculated. Meanwhile, the fruit morphology was recorded using a camera (Canon PowerShot SX50 HS, Oita Prefecture, Japan).

### 4.3. Kernel Oil Content and Fatty Acid Composition Detection

The walnut seed kernels dried to constant weight were ground to a homogenized powder. Then, 1.50 g of the powder was put in a water-free and oil-free filter paper bag used to extract crude fat using a Soxhlet apparatus at 75 °C for 8 h with anhydrous ether (boiling point 34.5 °C) as the extractant. Fatty acid methyl esters (FAMEs) were assayed by gas chromatography with flame ionization detection (GC–FID) and DB-23 chromatographic column (Agilent Technologies, Santa Clara, CA, USA) using the method described by Poggetti et al. [16]. Relative percentage of FAs was calculated according to the peak areas. All analyses were carried out in triplicate. The data were expressed as mean ± standard error (SE).

### 4.4. Total RNA Isolation, Transcriptome Sequencing Library Construction, and the Next Generation Sequencing

The total RNA of the walnut kernel samples was extracted using a Plant RNA Kit (OMEGA Bio-Tek, Norcross, GA, USA) according to the instructions. Three methods were used to detect RNA quality and quantity: agarose gel electrophoresis was used to detect the RNA degradation and contamination, the Nano Photometer^®^ spectro photometer (IMPLEN, Munich, Germany) was used to check RNA purity, and the Bioanalyzer 2100 system (Agilent Technologies, CA, USA) was used to check RNA integrity. A transcriptome sequencing library was constructed using a NEBNext^®^ Ultra^TM^ RNA Library Prep Kit for Illumina^®^ (Illumina, San Diego, CA, USA) following the producer’s recommended technology process. The library quality was assessed on the Agilent Bioanalyzer 2100 system. Illumina sequencing was performed at Novogene Bioinformatics Technology Co., Ltd., Beijing, China. The library preparations were sequenced on the Illumina Novaseq 6000 platform and 150 bp paired-end reads were generated.

### 4.5. Analysis of Transcriptome, Quality Control and Clean Reads Mapping to the Reference Genome

All sequencing results were assessed for quality control by removing reads containing adapter, reads containing ploy-N, and low-quality reads from the raw data, and thus we obtained clean data that were used for downstream analyses. All clean data were mapped to the walnut genome reference sequence (GenBank assembly accession: GCF_001411555.1) using Hisat2 v2.0.5 [55].

### 4.6. Quantification of Gene Expression Level and Differential Expression Analysis

The reads numbers mapped to each gene were counted using feature Counts v1.5.0-p3 [56]. FPKM values for each gene were calculated based on the length of the gene and reads count were mapped to this gene, and were used for estimating gene expression levels [57]. Differential expression analysis of five groups (three biological replicates per group) was performed using the DESeq2 R package (1.16.1) [58]. The Benjamini and Hochberg’s approach was used to adjust the resulting p-values for controlling the false discovery rate [59]. Genes with an adjusted *p*-value < 0.05 found by DESeq2 were said to be differentially expressed.

### 4.7. GO and KEGG Enrichment of DEGs

The clusterProfiler R package was used to analyze the DEGs and correct the gene length bias. GO terms with corrected *p*-values less than 0.05 were considered significantly enriched by DEGs. The clusterProfiler R package was used to test the statistical enrichment of DEGs in KEGG pathways [60].

### 4.8. WGCNA

The gene coexpression network was constructed by WGCNA of NovoMagic Cloud platform accessed on 6 June 2022 (https://magic.novogene.com/). The network diagram was created using Cytoscape 3.9.1 accessed on 26 June 2022 (https://cytoscape.org/).

### 4.9. Quantitative Real-Time PCR and Correlation Analyses

The total RNA was extracted using a TaKaRa MiniBEST Plant RNA Extraction Kit according to the manufacturer’s instructions, and cDNA was synthesized from the total RNA (500 ng) using TaKaRa PrimeScript ^TM^ RT Master Mix (Takara Biotechnology Co., Ltd., Dalian, China) to reach 10 μL total volume following the instructions (Takara). The qRT-PCR was performed with TaKaRa TB Green^®^ Premix Ex Taq^TM^ II (Tli RNaseH Plus) (Takara Biotechnology Co., Ltd., Dalian, China) according to the manufacturer’s instructions, and was performed on a CFX Connect ^TM^ Real-Time System (Bio-Rad Laboratories, CA, USA). As the housekeeping gene, walnut *GAPDH* was used as the reference gene [29]. The primers for qRT-PCR were designed with Oligo software (Table S5). The relative expression level of the target genes was calculated by 2^−ΔΔCT^ method [61]. Pearson correlation analysis of the expression levels between qRT-PCR and RNA-seq was conducted by GraphPad Prism 6.0., and *p* ≤ 0.01 was the threshold for statistical significance (**).

## 5. Conclusions

In this study, the oil contents of *J. regia* seed kernels from the Qinghai Plateau in different developmental stages were measured. The results indicated that walnut oils increased dramatically in late July and reached the maximum value of 69% in mature seeds. The 18:2 was the predominant UFA accumulated in mature seeds. The transcriptome of *J. regia* at five developmental stages was sequenced and annotated using the Illumina RNA-seq technology. Through transcriptomics profiling, four sets of DEGs in the different seed development stages were obtained. Compared to G1, the number of DEGs increased with the seed kernel development. The DEGs related to lipid biosynthesis and metabolism were screened by the DESeq method. We counted the number of DEGs associated with the lipid metabolic and oil accumulation. The key regulatory enzymes were identified, and they may play crucial roles in FA biosynthesis and oil accumulation in walnut seed kernels. FA de novo biosynthesis-related genes, including *ACCase*, *MAT*, *KAS*, *KAR*, *HAD*, *EAR*, *FATB* and *FATA*, were highly expressed from G1 to G4 stage. *LACS*, *KCS*, *KCR*, *HCD*, *ECR*, *SAD*, and *FADs* were related to FA elongation and desaturation. *GPAT*, *LPLAT*, *PAP*, *PLA2*, *DGAT*, *PDAT*, and *PDCT* were involved in TAG assembly pathway. The synergy of these genes promoted oil synthesis, and highly expressed oleosins, caleosins and steroleosins may be important for timely energy reserve in oil bodies. This transcriptome data will enrich public databases and provide new insights into functional genes related to the seed kernel lipid metabolism and oil accumulation in *J. regia.* Heat shock protein and heat stress TFs may protect walnuts from damage caused by large temperature differences and provide a guarantee for high lipid content. WGCNA showed that *AP2/ERF* and *bHLH* were the key TFs in lipid biosynthesis in walnut seed kernels, and were co-expressed with *ACC1*, *α-CT*, *BCCP*, *MAT*, *KASII*, *LACS*, *FATA*, and *PDCT*. Our results will serve as a basis to investigate regulation networks of *J. regia* to clarify the molecular mechanisms of oil accumulation process and to accelerate the genetic engineering to increase seed oil content and quality. Our results may also provide a useful reference for the molecular breeding of woody oil plants.

## Figures and Tables

**Table 1:** The fatty acid composition and content in mature seed kernels (%, *w/w*).

Fatty Acid Type			Content
Saturated (SFA)		Myristic acid (14:0)	0.0220 ± 0.0007
		Palmitic acid (16:0)	6.4067 ± 0.1109
		Heptadecanoic acid (17:0)	0.0539 ± 0.0007
		Stearic acid (18:0)	2.0400 ± 0.0829
		Arachidic acid (20:0)	0.0863 ± 0.0075
		Docosanoic acid (22:0)	0.0429 ± 0.0069
		Tetracosanoic acid (24:0)	0.0195 ± 0.0138
Unsaturated (UFA)	Monounsaturated (MUFA)	Palmitoleic acid (16:1)	0.1217 ± 0.0074
		Heptadecenoic acid (17:1)	0.0320 ± 0.0023
		Oleic acid (18:1)	19.9333 ± 0.4190
		11-Eicosenoic acid (20:1)	0.1753 ± 0.0146
		Erucic acid (22:1)	0.0270 ± 0.0259
		Nervonic acid (24:1)	0.0150 ± 0.0111
	Polyunsaturated (PUFA)	Linoleic acid (18:2)	57.6667 ± 0.7717
		Linolenic acid (18:3)	13.3333 ± 0.2867
		Eicosadienoic acid (20:2)	0.0261 ± 0.0015
SFA			8.6713 ± 0.1688
UFA			91.3304 ± 0.1675
MUFA			20.3043 ± 0.4147
PUFA			71.0261 ± 0.5715
MUFA/SFA			2.3417 ± 0.0296
PUFA/SFA			8.1952 ± 0.2219
MUFA/PUFA			0.2859 ± 0.0081

**Table 2:** The numbers of the DEGs in 15 lipid metabolism KEGG pathways.

KEGGID	Description	Stage	GeneRatio	Count	Up	Down
ath00061	Fatty acid biosynthesis	G2 vs. G1	10/853	10	3	7
		G3 vs. G1	27/1735	27	11	16
		G4 vs. G1	37/2191	37	8	29
		G5 vs. G1	50/3080	50	6	44
ath00062	Fatty acid elongation	G2 vs. G1	7/853	7	2	5
		G3 vs. G1	15/1735	15	4	11
		G4 vs. G1	17/2191	17	5	12
		G5 vs. G1	23/3080	23	9	14
ath00100	Steroid biosynthesis	G2 vs. G1	14/853	14	4	10
		G3 vs. G1	21/1735	21	6	15
		G4 vs. G1	26/2191	26	6	20
		G5 vs. G1	35/3080	35	8	27
ath00561	Glycerolipid metabolism	G2 vs. G1	21/853	21	10	11
		G3 vs. G1	29/1735	29	14	15
		G4 vs. G1	35/2191	35	14	21
		G5 vs. G1	44/3080	44	20	24
ath00564	Glycerophospholipid metabolism	G2 vs. G1	19/853	19	9	10
		G3 vs. G1	37/1735	37	15	22
		G4 vs. G1	46/2191	46	18	28
		G5 vs. G1	60/3080	60	25	35
ath00590	Arachidonic acid metabolism	G2 vs. G1	6/853	6	4	2
		G3 vs. G1	9/1735	9	5	4
		G4 vs. G1	10/2191	10	7	3
		G5 vs. G1	16/3080	16	11	5
ath00592	alpha-Linolenic acid metabolism	G2 vs. G1	11/853	11	7	4
		G3 vs. G1	21/1735	21	11	10
		G4 vs. G1	21/2191	21	13	8
		G5 vs. G1	33/3080	33	19	14
ath01040	Biosynthesis of unsaturated fatty acids	G2 vs. G1	7/853	7	3	4
		G3 vs. G1	15/1735	15	7	8
		G4 vs. G1	13/2191	13	6	7
		G5 vs. G1	18/3080	18	5	13
ko00071	Fatty acid degradation	G2 vs. G1	16/853	16	6	10
		G3 vs. G1	26/1735	26	10	16
		G4 vs. G1	29/2191	29	11	18
		G5 vs. G1	43/3080	43	22	21
ko00591	Linoleic acid metabolism	G2 vs. G1	2/853	2	2	0
		G3 vs. G1	2/1735	2	2	0
		G4 vs. G1	4/2191	4	3	1
		G5 vs. G1	7/3080	7	5	2
ko00600	Sphingolipid metabolism	G2 vs. G1	2/853	2	0	2
		G3 vs. G1	7/1735	7	1	6
		G4 vs. G1	10/2191	10	4	6
		G5 vs. G1	14/3080	14	7	7
ko00073	Cutin, suberine and wax biosynthesis	G2 vs. G1	2/853	2	1	1
		G3 vs. G1	5/1735	5	3	2
		G4 vs. G1	5/2191	5	2	3
		G5 vs. G1	7/3080	7	4	3
ko00565	Ether lipid metabolism	G2 vs. G1	6/853	6	2	4
		G3 vs. G1	9/1735	9	1	8
		G4 vs. G1	14/2191	14	5	9
		G5 vs. G1	21/3080	21	8	13
ko00072	Synthesis and degradation of ketone bodies	G2 vs. G1	2/853	2	2	0
		G3 vs. G1	4/1735	4	2	2
		G4 vs. G1	4/2191	4	2	2
		G5 vs. G1	6/3080	6	3	3
ko01212	Fatty acid metabolism	G2 vs. G1	18/853	18	6	12
		G3 vs. G1	43/1735	43	17	26
		G4 vs. G1	50/2191	50	13	37
		G5 vs. G1	69/3080	69	17	52

Note: Up: upregulated; DOWN: downregulated; GeneRatio: the ratio of the number of differential genes on the GO number to the total number of differential genes.

## Data Availability

Transcriptome sequencing data are available from the NCBI under project ID PRJNA781571.

## Supplementary Materials

The following supporting information can be downloaded at: https://www.mdpi.com/article/10.3390/plants11233207/s1, Figure S1: Distribution of reads in different regions of the genome at five developmental stages, Figure S2: Gene ontology classification of the walnut transcriptome data, Figure S3: Bubble diagram of enrichment of KEGG pathway, Figure S4: Temperature variation in the process of walnut seed development, Figure S5: Weighted gene coexpression network analysis of genes and the expression of TF at different walnut seed development stages; Table S1: The morphological and physiological characteristics, oil accumulation in the process of walnut embryo development, Table S2: The mapping result of each sample, Table S3. Distribution of sequencing reads in genome regions, Table S4: Detailed information regarding gene annotation and FPKM values of genes in developing walnut seeds, Table S5: The designed primers of the key enzymes involved in lipid biosynthesis and metabolism for qRT-PCR, Table S6: Identification of transcription factors in the transcriptome of *J. regia.* in blue module.
