# Supplementary material for: Transcriptome Analysis of Genes Involved in Fatty Acid and Lipid Biosynthesis in Developing Walnut (Juglans regia L.) Seed Kernels from Qinghai Plateau
Source: Plants (Basel). 2022 Nov 23;11(23):3207. doi: 10.3390/plants11233207 (PMC9737478; doi:10.3390/plants11233207)
Supplement: Supplementary file 1 [file plants-11-03207-s001.zip › supplementary material/Figure S1-5/Caption Figure S1-S5.pdf]

Figure S1. Distribution of reads in different regions of the genome at five developmental stages. Fruit stage G1: G11, G12, G13; Fruit stage G2: G21, G22, G23; Fruit stage G3: G31, G32, G33; Fruit stage G4: G41, G42, G43; Fruit stage G5: G51, G52, G53.

Figure S2. Gene ontology classification of the walnut transcriptome data. Clustering analysis diagram of significantly enriched GO terms in different combinations: G2 vs. G1 (A), G3 vs. G1(B), G4 vs. G1(C), G5 vs. G1(D).

Figure S3. Bubble diagram of enrichment of KEGG pathway. The KEGG pathways related to fatty acid biosynthesis and lipid metabolism of G2 vs. G1 (A), G3 vs. G1(B), G4 vs. G1 (C), G5 vs. G1 (D), accordingly.

Figure S4. Temperature variation in the process of walnut seed development.

Figure S5. (A) Gene numbers of 35 modules . (B) Module-trait relationships using WGCNA. Each column corresponds a specific stage and each row corresponds to a module eigengene. Each cell contains the corresponding correlation (top number) and p-value (bottom number). (C) Heatmap of eigengene expression in blue module. (D) The expression levels of *AP2/ERF* and *bHLH* during five developmental stages.
