# Supplementary figures and images for: Transcriptome Analysis of Genes Involved in Fatty Acid and Lipid Biosynthesis in Developing Walnut (Juglans regia L.) Seed Kernels from Qinghai Plateau
Source: Plants (Basel). 2022 Nov 23;11(23):3207. doi: 10.3390/plants11233207 (PMC9737478; doi:10.3390/plants11233207)

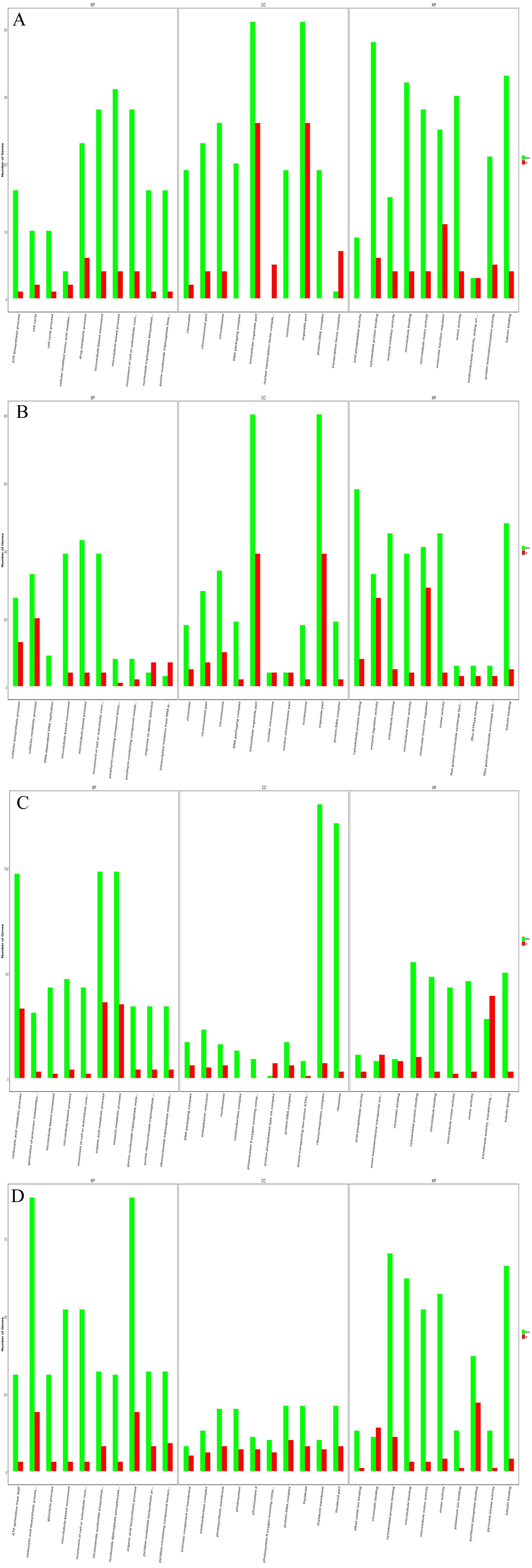

Supplement: Supplementary file 1 [file plants-11-03207-s001.zip › supplementary material/Figure S1-5/Figure S2.tif]

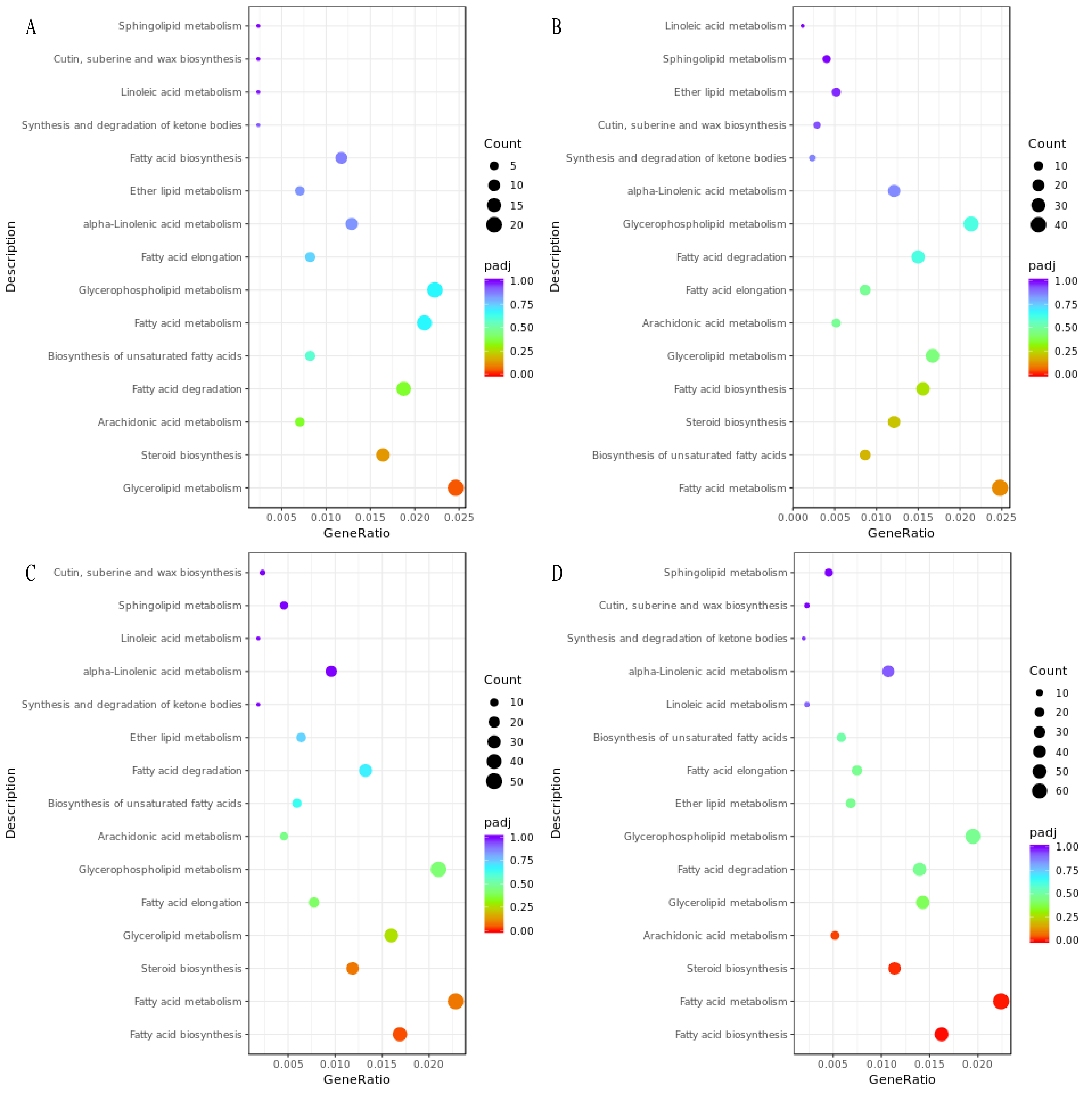

Supplement: Supplementary file 1 [file plants-11-03207-s001.zip › supplementary material/Figure S1-5/Figure S3.jpg]

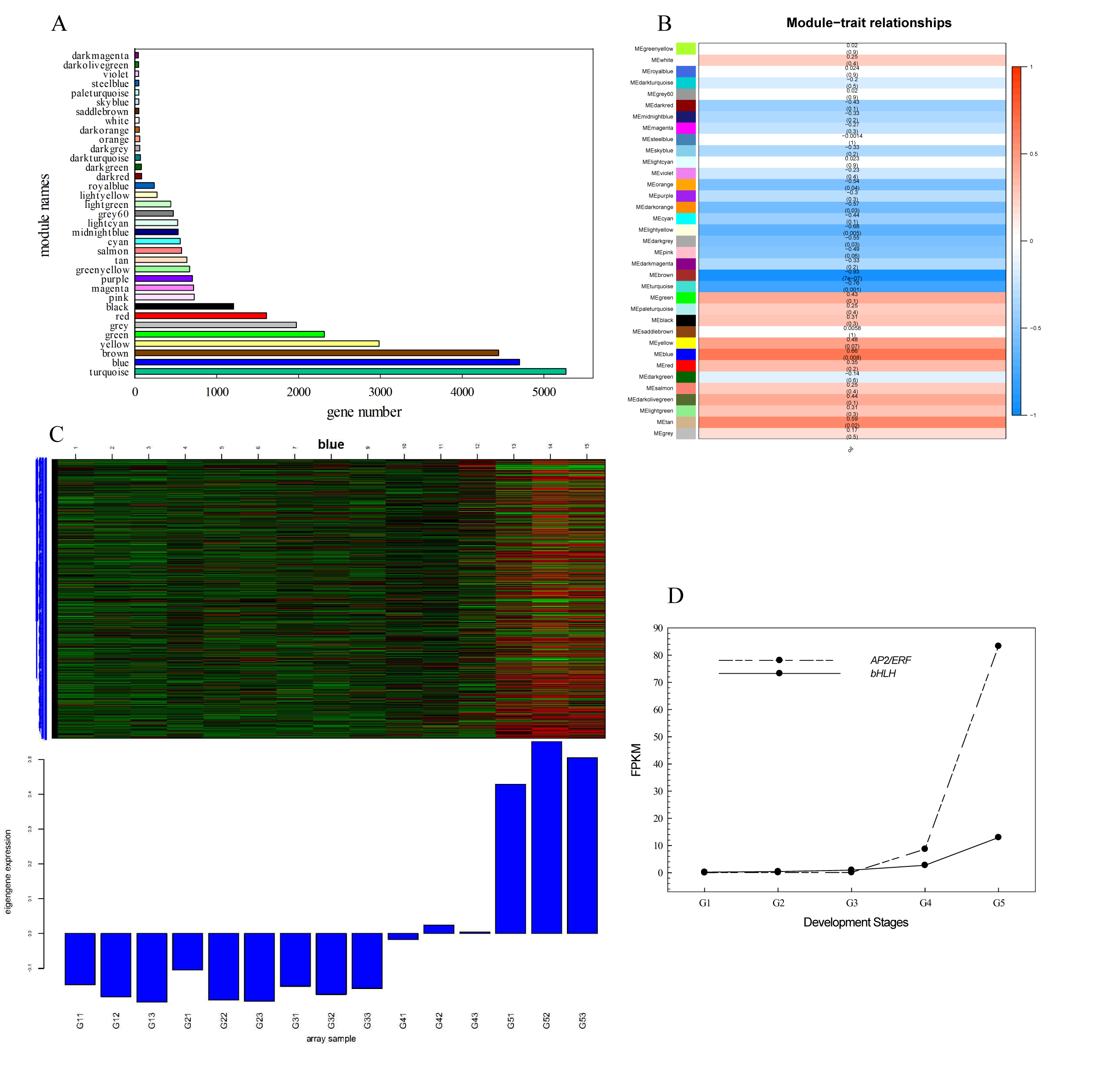

Supplement: Supplementary file 1 [file plants-11-03207-s001.zip › supplementary material/Figure S1-5/Figure S5.tif]
